# New Insight into Intrachromosomal Deletions Induced by Chrysotile in the *gpt* delta Transgenic Mutation Assay

**DOI:** 10.1289/ehp.9425

**Published:** 2006-09-06

**Authors:** An Xu, Lubomir B. Smilenov, Peng He, Ken-ichi Masumura, Takehiko Nohmi, Zengliang Yu, Tom K. Hei

**Affiliations:** 1 Center for Radiological Research, College of Physicians & Surgeons, Columbia University, New York, New York, USA; 2 Key Laboratory of Ion Beam Bioengineering, Institute of Plasma Physics, Chinese Academy of Sciences, Hefei, Anhui, People’s Republic of China; 3 Division of Genetics and Mutagenesis, National Institute of Health Sciences, Tokyo, Japan; 4 Department of Environmental Health Sciences, Mailman School of Public Health, Columbia University, New York, New York, USA

**Keywords:** chrysotile asbestos, *gpt* delta transgenic mutation system, kilobase-sized mutation, oxyradicals, γ-H2AX

## Abstract

**Background:**

Genotoxicity is often a prerequisite to the development of malignancy. Considerable evidence has shown that exposure to asbestos fibers results in the generation of chromosomal aberrations and multilocus mutations using various *in vitro* approaches. However, there is less evidence to demonstrate the contribution of deletions to the mutagenicity of asbestos fibers *in vivo*.

**Objectives:**

In the present study, we investigated the mutant fractions and the patterns induced by chrysotile fibers in *gpt* delta transgenic mouse primary embryo fibroblasts (MEFs) and compared the results obtained with hydrogen peroxide (H_2_O_2_) in an attempt to illustrate the role of oxyradicals in fiber mutagenesis.

**Results:**

Chrysotile fibers induced a dose-dependent increase in mutation yield at the *redBA/gam* loci in transgenic MEF cells. The number of λ mutants losing both *redBA* and *gam* loci induced by chrysotiles at a dose of 1 μg/cm^2^ increased by > 5-fold relative to nontreated controls (*p* < 0.005). Mutation spectra analyses showed that the ratio of λ mutants losing the *redBA*/*gam* region induced by chrysotiles was similar to those induced by equitoxic doses of H_2_O_2_. Moreover, treatment with catalase abrogated the accumulation of γ-H2AX, a biomarker of DNA double-strand breaks, induced by chrysotile fibers.

**Conclusions:**

Our results provide novel information on the frequencies and types of mutations induced by asbestos fibers in the *gpt* delta transgenic mouse mutagenic assay, which shows great promise for evaluating fiber/particle mutagenicity *in vivo*.

Asbestos fibers, a group of naturally occurring silicate minerals, are well-established human carcinogens. They are causally related to the development of asbestosis, bronchial carcinoma, malignant mesothelioma of the pleura and peritoneum, and possibly cancers of the gastrointestinal tract and larynx (Gustavsson et al. 1998; International Agency for Research on Cancer 1987). Although the main concern of asbestos-related diseases focuses primarily on the work-place, the danger of developing such diseases now extends beyond that of a simple occupational hazard because accumulating evidence suggests that asbestos fibers are widely distributed in the environment to which the general public may be exposed (Gardner and Saracci 1989). Individuals may be subjected to prolonged exposure to asbestos in their homes, schools, drinking water, neighborhoods of industrial sources of asbestos, or areas of a natural occurring asbestos (Kjaerheim et al. 2005; Miller 2005; Rom et al. 2001). Recent evidence indicates an excess risk of mesothelioma in individuals living in the vicinity of a natural occurring source of asbestos (Pan et al. 2005). The continued discovery of routes through which the general public may be exposed to asbestos suggests a long-term, low-level exposure of a large number of people, which may lead to an elevated risk of asbestos-related diseases.

The mechanisms by which asbestos produces malignancy are unclear at present. It has been reported that fiber dimension, biopersistence, composition, surface reactivity, and physical durability are important criteria for the carcinogenicity of the fibers, indicating that carcinogenic mechanisms of asbestos are likely to be complex and involve multiple pathways (Bernstein et al. 2003; Coin et al. 1994). Various highly quantitative genotoxicity assays ranging from DNA strand breaks to gene mutations in rodent cells have been performed to estimate the carcinogenic potential of asbestos fibers (Lezon-Geyda et al. 1996; Poser et al. 2004). Although asbestos fibers have been shown to induce chromosomal aberrations and sister chromatid exchanges, mutagenic studies at the hypoxanthine–guanine phosphoribosyl transferase (*hprt*; GenBank accession no. NM_013556; http://www.ncbi.nlm.nih.gov/) and ouabain loci in mammalian cells have been shown to be either inactive or weakly active (Jaurand 1996). Using the human–hamster hybrid cell line (A_L_) in which mutations are scored at a marker gene (*CD59*) located on human chromosome 11 (11p13) that the A_L_ cell carries as its only human chromosome, our previous studies have demonstrated that asbestos fibers are indeed mutagenic and induce mostly deletions involving millions of base pairs (Hei et al. 1992). In contrast, among the same fiber-treated A_L_ cell population, there are few, if any, mutations scored at the *hprt* locus of the hamster X chromosome. This discrepancy has been attributed to the possibility that the *hprt* gene is located on the X chromosome, and large deletions in the region of the gene that are required for cell survival would be lethal and any mutants induced would not be viable. In recent years several other mutagenic assays that are proficient in detecting either large deletions, homologous recombinations, or score mutants located in nonessential genes have been used successfully to demonstrate the mutagenic potential of various fiber types (Lezon-Geyda et al. 1996; Park and Aust 1998). These findings provide a direct link between chromosomal abnormalities that frequently have been demonstrated in fiber exposed human and rodent cell lines and carcinogenicity *in vivo*. However, there is less direct evidence that illustrates chromosomal mutations of asbestos fibers in various organs and tissues in intact organisms.

The use of transgenic mouse systems carrying bacterial reporter genes such as *lacZ*, *lacI*, and *cII* has opened a promising opportunity for short-term mutagenicity analysis (Dean et al. 1999). There is evidence to show that asbestos fibers are mutagenic and induce point mutations in either Big Blue transgenic mice or rats bearing λ-*lacI* as a reporter gene (Rihn et al. 2000; Topinka et al. 2004; Unfried et al. 2002). However, most genome-wide mutations such as large deletions, insertions, translocations, and aneuploidy are not effectively recovered by the *lacI* shuttle vector. To efficiently recover large deletions *in vivo*, *gpt* (xanthine phosphoribosyltransferase; GenBank accession no. NP_414773; http://www.ncbi.nlm.nih.gov/GenBank) delta transgenic mice have been established by integrating multiple copies of λ EG10 DNA with the *redBA* and *gam* (Genbank accession no. J02459; http://www.ncbi.nlm.nih.gov/GenBank) genes into each chromosome 17 of C57BL/6J mice (Nohmi and Masumura 2004). Because wild-type λ-phage DNA replicate poorly in the presence of P2 lysogens in the host cells (called “sensitive to P2 interference” or “Spi”), only mutant λ phages that are deficient in the functions of both the *redBA* and *gam* genes are able to escape from P2 interference (called “Spi^−^”) and form visible clear plaques on a bacterial lawn. Simultaneous inactivations of both the *redBA* and *gam* genes, an indication of deletions in the gene loci region, provide an available method to quantify deletion mutations induced by various physical and chemical mutagens, such as X rays and alkylating agents (Horiguchi et al. 2001; Shibata et al. 2005).

Chrysotile asbestos, a fibrous serpentine, is the most commercially used form of asbestos in the world trade and accounts for > 95% of asbestos found in United States buildings. In the present study we adapted the *gpt* delta transgenic mouse mutation system to evaluate the genotoxicity of chrysotile in *gpt* delta mouse primary embryo fibroblast (MEF) cells. We investigated the mutation frequencies at both *redBA* and *gam* (GenBank accession no. J02459; http://www.ncbi.nlm.nih.gov/GenBank) loci and the contribution of deletions > 2 kb to the mutagenicity of chrysotile fibers. Because reactive oxygen species (ROS) such as superoxide anions (O_2_^−^) and hydrogen peroxide (H_2_O_2_) originate not only from redox reactions catalyzed on the fiber surface but also from the incomplete phagocytosis of fibers in various cells, such as phagocytic, mesothelial, and rat lung epithelium cells, we speculated that asbestos fibers would induce similar types of mutations as that of chemically generated oxyradicals. We found that both chrysotile and H_2_O_2_ dramatically increased the mutation yield, which could be abrogated by concurrent treatment with catalase. Furthermore, the ratios of mutants with deletions > 2 kb were similar to those generated by oxyradicals at two equitoxic doses. The accumulation of phosphorylated histone H2AX (γ-H2AX) further demonstrated the involvement of DNA double-strand break (DSB) in the mutagenicity of chrysotiles. These results provide direct evidence that asbestos fibers induced kilobase pair deletion mutations in a transgenic mouse mutation system, and that these were mediated by oxyradicals.

## Materials and Methods

### MEF cell culture

*gpt* delta transgenic mice, obtained from T. Nohmi, were mated, and pregnant females were sacrificed on day 14 of the experimental protocol were previously approved by the Columbia University Institutional Animal Care and Use Committee. The animals were treated humanely and with regard for the alleviation of pain and suffering. The embryos were surgically removed and embryonic tissue prepared in culture according to standard procedures (Hogan et al. 1994). These cultures were grown and maintained in Dulbecco’s modified Eagle’s medium (Gibco-BRL, Gaithersburg, MD, USA) containing 15% heat-inactivated fetal bovine serum and penicillin (100 U/mL), streptomycin (50 μg/mL) in a 5% CO_2_ environment at 37°C.

### Chrysotile preparation

We used International Union Against Cancer standard reference chrysotile asbestos (average length, 7.8 μm; average diameter, 0.2 μm) in these studies (Timbrell 1979). The fibers were prepared as described previously (Hei et al. 1992). Briefly, samples of fibers were weighed and suspended in distilled water. The fiber suspension was triturated 6–8 times with a 20-gauge syringe needle. A stock solution of the fibers was sterilized by autoclaving and mixed to ensure a uniform suspension before being diluted with tissue culture medium for cell treatment.

### Cytotoxicity assay

We evaluated cell viability using MTT (3-(4,5-dimethylthiazol-2-yl)-2,5-diphenyltetrazolium bromide) assay on the basis of the ability of viable cells to convert a water-soluble tetrazolium salt into a water-insoluble formazan product (Scudiero et al. 1988). The enzymatic reduction of the tetrazolium salt happens only in living, metabolically active cells but not in dead cells. Cultures were incubated in two-well chamber slides at a density of 1.0 × 10^5^ cells per well at 37°C for 24 hr. Graded doses of either chrysotile or H_2_O_2_ were added to the culture medium and incubated for another 24 hr in the presence of serum (chrysotile) or 15 min in the absence of serum (H_2_O_2_). At the end of the treatment period, the medium was removed, 200 μL of 5 mg/mL MTT was added to each well, and the cultures were incubated for another 4 hr. The supernatant was removed and 1 mL acidic isopropanol was added to dissolve the formazan crystals. The absorbance at 570 nm was determined by an Ultrospec 3100 pro UV/Visible spectrophotometer (Biochrom Ltd., Cambridge, UK).

### Genomic DNA isolation

We isolated genomic DNA from MEF cells using the RecoverEase DNA isolation kit (Stratagene, La Jolla, CA, USA) according to the protocol developed by the supplier. Briefly, about 5.0 × 10^6^ cells were transferred to a chilled Wheaton dounce tissue grinder (Fisher, Hampton, NH, USA), and the homogenate obtained was filtered and centrifuged at 1,100 × *g* for 12 min at 4°C. The pellet was resuspended in digestion buffer containing RNAses (RANse-It; Stratagene) containing proteinase K solution (2 mg/mL prewarmed to 50°C). Using wide-bore pipette tips, the samples were transferred to dialysis cups floating on the surface of TE buffer (500 mL) and dialyzed for 24 hr. The purity and concentration of DNA was checked spectrophotometrically and samples were diluted with TE [10 mM Tris–Cl (pH 7.5), 1 mM EDTA] buffer to a final DNA concentration of approximately 0.5 mg/mL, and stored at 4°C for up to 3 months prior to mutation analysis.

### In vitro *packaging of DNA.*

The λ DNA was recovered from approximately 5 μg genomic DNA and packaged with terminase and phage proteins contained in the Transpack kit (Stratagene) to produce infectious λ phages. Viable phages were infected into *Escherichia coli* XL-1 Blue MRA (Stratagene), mixed with λ-trypticase agarose and poured onto 100-mm plates containing 30 mL bottom agar. Plates were incubated overnight at 37°C. The average of rescued phages per packaging reaction was 1.8 × 10^6^ in the present studies. There was no significant difference in the titers between control and exposed groups.

### Spi^−^ mutation analysis

The mutant frequencies at *red/gam* loci were determined by Spi^−^ selection as described previously (Nohmi and Masumura 2004; Nohmi et al. 1996; Shibata et al. 2005*)*. Briefly, packaged phages were infected into *E. coli* XL-1 Blue MRA (P2) (Stratagene). Infected cells were mixed with molten soft agar, poured onto λ-trypticase agar plates and incubated at 37°C. The plaques detected on the plates (Spi^−^ candidates) were suspended in 50 μL of SM buffer [0.58% NaCl, 0.2% MgSO_4_ · 7H_2_O, 50 mM Tris–HCl, 0.01% gelatin (pH 7.5)]. The suspension was spotted on the two types of plates where *E. coli* XL-1 Blue MRA (P2) or WL95 (P2) strain was spread. The plates were incubated for 24 hr at 37°C. The numbers of mutants that made clear spots on both strains were counted as confirmed Spi^−^ mutants. Mutation frequencies were calculated by comparing the titration and number of confirmed mutant plaques.

### Spi^−^ mutant characterization

To determine the mutated region, the phage DNA was used and subjected to DNA sequence and polymerase chain reaction (PCR) analysis with various sets of primers (Horiguchi et al. 2001). The PCR primers used were as follows: primer 1: 5′-CACTCTCTTTGATGCGAATGCCAGCGTCAGAC-3′; primer 2: 5′-CAGGAGTAATTATGCGGAACAGAATCATGCCTGGTG-3′; primer 3: 5′-GTGAGGATGCGTCATCGCCATTGCTCCCC-3′; primer 4: 5′-GCGATGAAAAGATGTTTCGTGAAGCCGTCGACGC-3′; primer 5: 5′-AAACAGGCGCGGGCATCAGCGTGGTCTGA-39′; primer 12: 5′-CGCGCGGTCGAGGGACCTAATAACTTCGTA-3′. We performed PCR amplification under the following conditions: 4 μL of phage DNA, 0.2 mM each dNTP, 1.5 mM MgCl_2_, Taq DNA polymerase (or ExTaq; Takara Shuzo Co., Kyoto, Japan), and 20 pmol of each primer in a 40-μL reaction volume; heating for 1–2 min at 94°C, and 24 cycles at 98°C for 20 sec and at 68°C for X minutes (1 min/1 kb), followed by final extension at 72°C for 10 min. The products were analyzed using agarose gel electrophoresis.

The PCR products were sequenced by ABI’s 3100 capillary sequencers (Dye Terminator Cycle Sequencing; PE Applied Biosystems, Foster City, CA, USA). PCR products for templates of sequence were purified using PCR product presequencing kit (Amersham Life Science, Piscataway, NJ, USA).

Sequence primers are as follows: s102: 5′-AATCCAAACTCTTTACCCGTCCTTGGGT-3′; s201: 5′-CGCTTGATAACTCTGTTGAATGGCTCT-3′; s301: 5′-GGTGGAATCCCATCAGCGTTACCGTTT-3′; s302: 5′-AGTGATTGCGCCTACCCGGATATTATCGTG-3′; s403: 5′-CCAGCCGACACGTTCAGCCAGCTTCCCAG-3′. The entire DNA sequence of λ EG10 is available at http://dgm2alpha.nihs.go.jp (Masumura et al. 2003).

### Determination of H2AX phosphorylation using flow cytometry

The cells were fixed by adding 2% paraformaldehyde dropwise while being vortexed. The fixed cells were then stained with mouse monoclonal anti–γ-H2AX (Upstate, Lake Placid, NY, USA) and fluorescein isothiocyanate (FITC)–conjugated secondary antibodies (Sigma-Aldrich Chemical Co., St. Louis, MO, USA) as described by Kurose et al. (2005). The cells were then suspended in 0.5 mL of 10 μg/mL propidium iodide (PI) and 40 μg/mL RNase A and incubated at 4°C for at least 30 min. The fluorescence of PI and FITC of individual cells induced by excitation with a 488-nm argon ion laser was measured using a FACSCalibur cytometer (BD Biosciences, San Jose, CA).

### Statistical analysis

All numerical data were calculated as mean and SD and evaluated by Student’s *t-*test. The statistical significance was tested at *p* < 0.05 as the critical value.

## Results

### Chrysotile-induced dose-dependent toxicity in transgenic MEF cells

**Figure 1:**
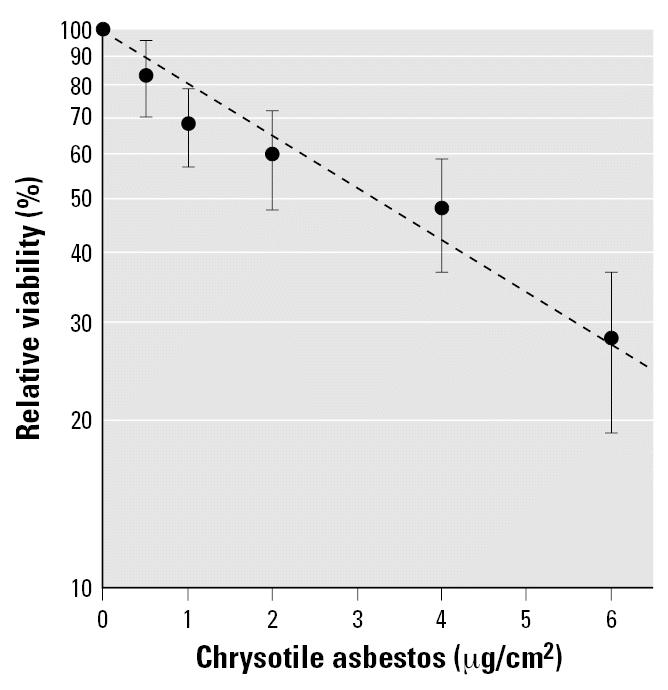
Cell viability of transgenic MEF cells treated with graded doses of chrysotile for 24 hr. Data were the average of three independent experiments. Error bars indicate ± SD.

The viability of MEF cells exposed to graded doses of chrysotile was analyzed by using the MTT assay. As shown in Figure 1, exposure of MEF cells to doses of chrysotiles ranging from 0.5 to 6 μg/cm^2^ for 24 hr produced a dose-dependent decrease in cell viability. The viabilities of MEF cells was reduced by 14, 29, and 59%, when the concentrations of chrysotile were 0.5, 1, and 2 μg/cm^2^, respectively. The median lethal dose of chrysotile, which resulted in 50% cell killing, was approximately 3.2 μg/cm^2^.

### *Mutation frequencies at* red/gam *gene loci were elevated in response to chrysotile exposure.*

**Figure 2:**
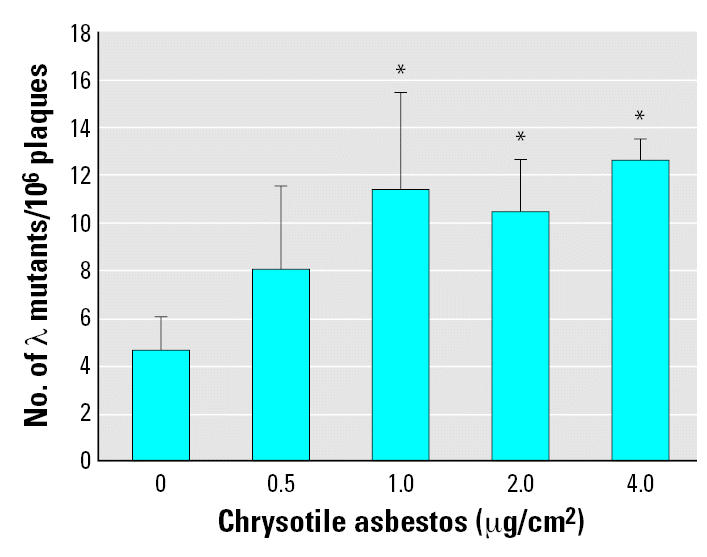
Mutagenic potential of chrysotile asbestos at *redBA* and *gam* loci in transgenic MEF cells. MEF cells, 5 × 10^5^, were treated with graded doses of chrysotile as described in the text. Results were expressed as the total number of confirmed λ mutants divided by the total number of rescued phages. The average number of preexisting mutants per 10^6^ plaques used for these experiments was 4.69 ± 1.42. Data were pooled from six independent experiments. Error bars indicate ± SD. *Significantly different at *p* < 0.05.

We have shown previously that asbestos is mutagenic and induces multilocus deletions in mammalian cells (Hei et al. 1992). To investigate the mutagenicity of asbestos in the *gpt* delta assay, we used an Spi^−^ mutation assay to determine the mutation frequencies induced by chrysotile exposure in transgenic MEF cells. The average number of spontaneous *red/gam* gene mutants per 10^6^ recovered plaques in MEF cells used for these experiments was 4.69 ± 1.80. Treatment of MEF cells with chrysotile fibers resulted in a dose-dependent induction of mutation yield at the *red/gam* gene locus (Figure 2). A significant increase in mutation yield over the background level was observed at fiber concentrations > 1 μg/cm^2^ (*p* < 0.005). The mutant fraction in cells treated with a dose of 1 μg/cm^2^ of fibers was 2.4-fold higher than background. These results indicated that chrysotile asbestos were able to produce deletion mutations in *gpt* delta transgenic mutation assay system.

### Characterization of mutant spectra induced by chrysotile

**Figure 3:**
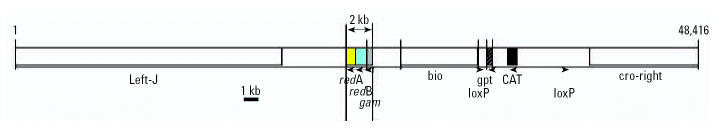
Schematic map of λ EG10 transgene. Abbreviations: bio, genetic marker used in bacteriophage lambda vectors; *CAT*, chloramphenicol acetyltransferase (GenBank accession no. AJ401050; http://www.ncbi.nlm.nih.gov/GenBank/); cro, transcription inhibitor; *gpt*, xanthine phosphoribosyltransferase (GenBank accession no. NP_414773); J, codes for phage tail gene; loxP, locus of X over P1, a site on the bacteriophage P1 consisting of 34 bp; *redA*, *redB*, and *gam*, single copy bacteriophage genes.

To determine the spectrum of mutations induced by chrysotile fibers, 93 and 74 λ mutants from control cells and cells treated with chrysotile at 1 μg/cm^2^, respectively, were subjected to either PCR analysis or DNA sequence analysis. The PCR product of *redBA/gam* in the wild-type λ EG10 was approximately 2 kb. If a PCR product did not show any discrete alteration on the gel, the mutant was classified as one containing a point mutation with either a base substitution or a frameshift causing no alteration in the size of the gene product. In contrast an absence of visible PCR product was taken as evidence of a mutant with a deletion > 2 kb as a result of losing both *redBA* and *gam* genes. The types of mutations identified from analysis of these mutants are listed in Table 1 and Figure 3. To minimize the possibility that these isolated mutants were spontaneously derived, we selected mutant phages from only the dose of chrysotile that resulted in the highest inductions over background levels. The majority of spontaneous mutants were deletions of various sizes throughout the *red BA/gam* genes (86 of 93 or 92%). Of these deletion mutants, 1 bp deletion made up 68 of 93 or 73%, whereas deletions ranging from 2 bp to 1 kb made up 8 of 93 or 8.6%. Of the spontaneous mutations with deletions 10 of 93 or 11% encompass regions of both the *gam* and *redBA* genes. In contrast, 41 of 74 or 56% and 10 of 74 or 14% of mutants recovered from chrysotile treated cells were single base pair deletion and deletions ranging from 2 bp to 1 kb, respectively. The proportion of mutants induced by chrysotile suffering loss of both the *gam* and *redBA* genes was increased from 10 of 93 or 11% among spontaneous mutants to 17 of 74 or 23% in fiber-treated MEF cells (Table 1).

### Deletions > 2 kb contribute to chrysotile-induced mutagenicity

To provide further evidence of the contribution of deletions > 2 kb to the mutagenicity of chrysotile, we compared the frequencies of deletions > 2 kb induced by chrysotile at a dose of 1 μg/cm^2^ with those derived spontaneously from control cultures (Table 2). Although the total Spi^−^ mutant yield in chrysotile-treated cells was 2.4-fold higher than that of controls, the frequency of deletions > 2 kb induced by 1 μg/cm^2^ of fibers was 5.2-fold higher than those derived from nontreated control (2.6 vs. 0.5 × 10^−6^, *p* < 0.005). The frequency of base substitution and small deletions including single base deletions and deletions < 1 kb formed in fiber-treated MEF cells was only 2-fold higher than those from nontreated cases. These results indicated that the major types of mutations induced by chrysotile were deletions > 2 kb.

### Oxyradicals mediated the mutagenicity of chrysotile in transgenic mouse mutation assay system

**Figure 4:**
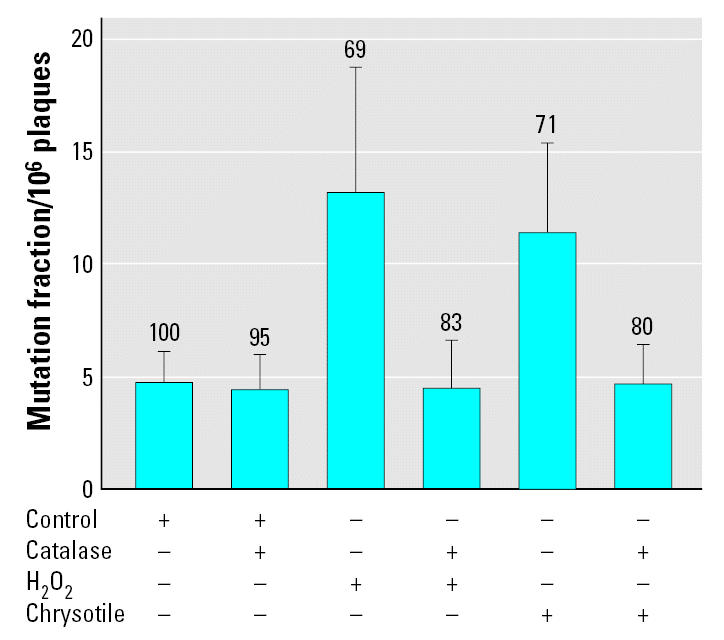
Mutant fractions at *redBA/gam* loci in MEF cells exposed to either chrysotile asbestos at a dose of 1 μg/cm^2^ or 2.9 mM H_2_O_2_ either in the presence (+) or absence (−) of catalase (5,000 U/mL). Results were expressed as the total number of confirmed λ-phage mutants divided by the total number of rescued phages. The average number of preexisting mutants per 10^6^ plaques used for these experiments was 4.69 ± 1.42. Numbers above error bars indicate the percentage of relative viability. Data were pooled from three to six independent experiments. Error bars indicate ± SD.

**Figure 5:**
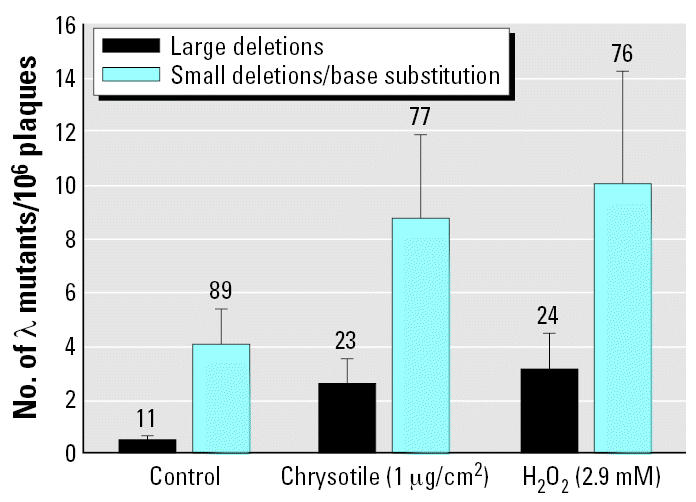
Mutant fractions of λ-phage mutants with specific molecular characteristics in MEF cells exposed to either chrysotile at a concentration of 1 μg/cm^2^ or H_2_O_2_ at a dose of 2.9 mM. Numbers above error bars indicate ratio of mutation type calculated as percentage. Data were pooled from three to six independent experiments. Error bars indicate ± SD.

There is evidence that the genotoxicity/carcinogenicity of asbestos fibers is mediated by reactive oxygen/nitrogen species (Shukla et al. 2003). To demonstrate that oxyradicals mediated the mutagenicity of chrysotile fibers in MEF cells, we exposed MEF cells to either chrysotile for 24 hr in complete medium, or to H_2_O_2_ in serum free medium for 15 min in the presence or absence of catalase (Figure 4). The relative viability of MEF cells treated with a 1 μg/cm^2^ dose of chrysotile was 71%, whereas the relative viability of MEF cells after exposing to 2.9 mM H_2_O_2_ was 69%. Both chrysotile and H_2_O_2_ led to significant increases in Spi^−^ mutant yields in MEF cells. As shown in Figure 4, the mutation yield induced by H_2_O_2_ treatment was slightly higher than that of chrysotile at equal toxic doses, although the difference was not statistically significant. Furthermore, the mutation yields induced by either chrysotile at a dose of 1 μg/cm^2^ or 2.9 mM H_2_O_2_ were dramatically suppressed in the presence of 5,000 U/mL catalase (*p* < 0.05). Interestingly, the ratio of the mutants with deletions > 2 kb was similar between chrysotile and H_2_O_2_ in that 20 of 84 or 24% of the mutants induced by 2.9 mM H_2_O_2_ lost both *redBA* and *gam* genes compared with 17 of 74 or 23% among those induced by a 1-μg/cm^2^ dose of chrysotile (Figure 5). The mutant fractions with deletions > 2 kb increased from 0.5 ± 0.16 observed in controls to either 2.6 ± 0.93 or 3.2 ± 1.34 in cells treated with either chrysotile or H_2_O_2_, respectively. The dose of catalase used here had little effect on the level of cell viability and mutant fraction in control cells. Similarly, heat-inactivated catalase (by boiling for 10 min) had little effect on the mutant fraction in exposed cells.

### Induction of γ-H2AX in MEF cells

**Figure 6:**
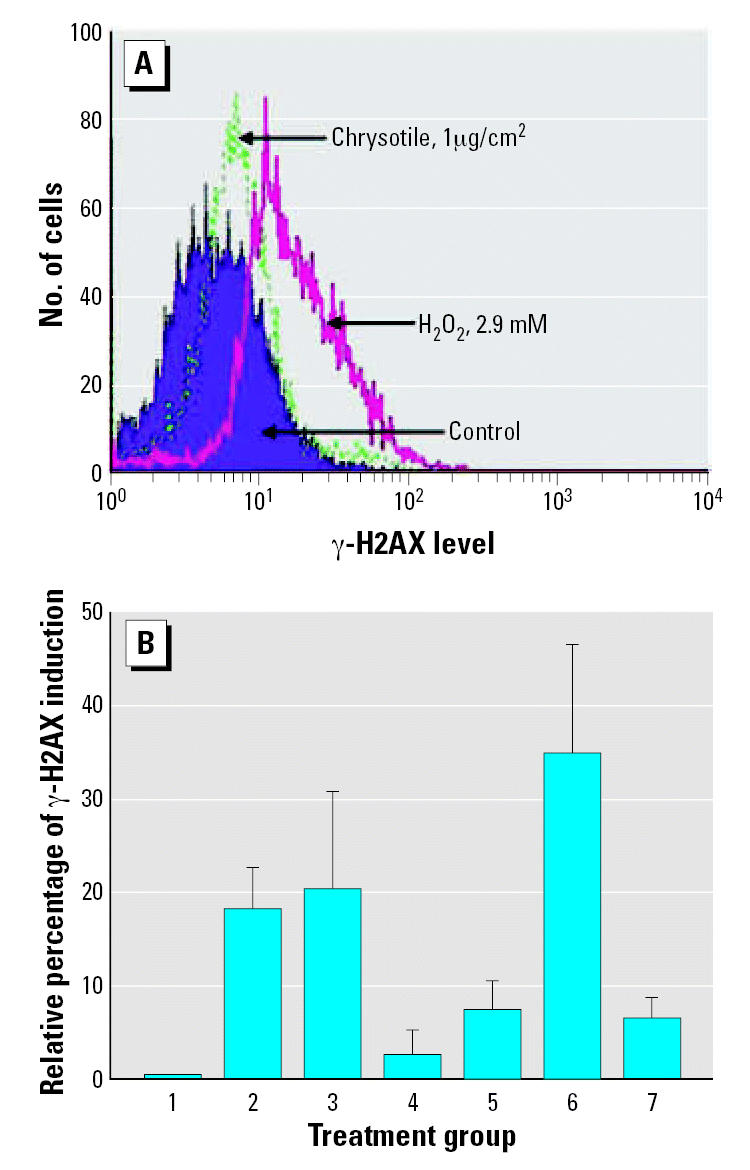
Accumulation of γ-H2AX in MEF cells exposed to either chrysotile for 24 hr in completed medium or H_2_O_2_ for 15 min in serum-free medium in the presence or absence of catalase. (*A*) Generation of γ-H2AX in cells treated with either chrysotile or H_2_O_2_ detected by flow cytometry using mouse monoclonal anti–γ-H2AX labeled with FITC. Results are shown using FL1-H. (*B*) Induced γ-H2AX in MEF cells exposed to either chrysotile or H_2_O_2_ in the presence or absence of catalase. (1) Control; (2) 1 μg/cm^2^ chrysotile; (3) 2 μg/cm^2^ chrysotile; (4) 5,000 U/mL catalase; (5) 1 μg/cm^2^ chrysotile + 5,000 U/mL catalase; (6) 29 mM hydrogen peroxide; (7) 29 mM hydrogen peroxide + 5,000 U/mL catalase. Data were pooled from three independent experiments. Error bars indicate ± SD.

Among various type of DNA damages, the DSBs in DNA may be the most damaging and genotoxic, which elevate the frequencies of gene translocations, rearrangements, amplifications, and deletions during repair and misre-pair of DSBs (Khanna and Jackson 2001). A very early step in the response of mammalian cells to DNA DSBs is the phosphorylation of histone H2AX at serine-139 at the sites of DNA damage. To investigate whether chrysotile induces phosphorylation of H2AX in MEF cells, we exposed cultures to either a 1- or 2-μg/cm^2^ dose of chrysotile for 24 hr before being fixed and stained with anti–γ-H2AX antibodies. The expression of phosphorylated H2AX as a function of DNA damage was then analyzed using flow cytometry. The histograms represented the frequency of cell number versus the intensity of the fluorescence signals of γ-H2AX antibody staining [FL1-H (green fluorescence signal received by the photomultiplier tube); Figure 6A]. Even though the number of foci/cell cannot be measured directly by flow cytometry, we found that MEF cells incubated with chrysotile showed an increased staining with anti–γ-H2AX antibodies as detected by immunofluorescence. However, there was no dose-dependent induction of γ-H2AX in MEF cells exposed to either 1 or 2 μg/cm^2^ doses of chrysotile (Figure 6B). Concurrent treatment of catalase greatly suppressed the induction of γ-H2AX among treated cells. These results suggest that chrysotile induced DNA damage that triggers a stress response leading to H2AX phosphorylation.

## Discussion

Asbestos fiber is an important environmental carcinogen worldwide and remains the primary occupational concern in many developing countries. Although the carcinogenicity of asbestos is well established, the underlying mechanism is not known. We have previously demonstrated that asbestos fibers are mutagenic and induce gene/chromosomal mutations in mammalian cells. Similar results have subsequently been reported by others using various *in vitro* and *in vivo* assays that can quantify multilocus deletions (Hei et al. 1992; Lezon-Geyda et al. 1996; Park and Aust 1998). However, it has not been established how asbestos fibers induce such mutational events *in vivo*.

Inhalation studies in Big Blue *lacI* transgenic mice have revealed that there is a 1.96-fold increase in mutation frequencies in lung tissues of crocidolite-exposed mice compared with nonexposed control mice, but no specific mutant spectrum has been identified (Rihn et al. 2000). More recently, mutation induction factors ranging from 1.1 to 3.2 in the omenta have been reported in Big Blue *lacI* transgenic rats injected with crocidolite (Unfried et al. 2002). Intratracheal instillation with amosite results in a 2-fold increase in the mutation frequency in lung DNA in Big Blue *lacI* transgenic rats (Topinka et al. 2004). It should be noted that the *lacI* transgenic system is limited to small sequence alterations between 1 and 20 bp, such as point mutations, small deletions, and insertions. Most genome mutations such as large deletions and insertions, translocations, and aneuploidy cannot be effectively recovered by the *lacI* shuttle vector. Several studies in which mutation frequencies in the *lacI* transgenic system were compared with that in endogenous genes have shown that spontaneous mutation frequencies at reporter genes were dramatically higher than those found at the endogenous *hprt* gene (Skopek et al. 1995; Walker et al. 1999). It is likely that overall mutagenesis induced by asbestos fibers may be underestimated in Big Blue *lacI* mice. As such, it is extremely desirable to establish an efficient system to recover large deletion events induced by asbestos fibers *in vivo*.

The *gpt* delta transgenic mouse system, established in the laboratory of one of the coauthors provides a unique opportunity to assess the *in vivo* mutagenic potential of mineral fibers (Masumura et al. 2003; Nohmi and Masumura 2004, 2005). The *gpt* delta mice carry tandem repeats of λ EG10 DNA in two units of 40 phage copies each on both arms of chromosome 17, which are retrievable as phage particles by an *in vitro* packaging reaction. The rescued phages are then used to quantify the mutation yield upon exposure to genotoxic agents. In the present study the MEF cells from the transgenic mice were used to both quantify and characterize the deletions induced by graded doses of chrysotile fibers. Our results demonstrated that chrysotiles induced a dose dependent increase in mutant yield at the *gam* and *redBA* loci in MEF cells and that the incidence and types of mutants generated were comparable to those induced by equitoxic doses of H_2_O_2_.

Among the mutants with deletions ≥ 2 kb that span the *redBA/gam* gene, the mutant fraction induced by treatment with a 1 μg/cm^2^ dose of chrysotiles was 5.2-fold higher than those derived spontaneously. The mutant fraction and the number of mutants with deletions > 2 kb, however, were not elevated by further increase in fiber doses. Although the precise reason for this lack of dose–response relationship is not clear, it is possible that mutated cells were selectively killed or that the λ phages were not effectively recovered *in vitro* at high fiber doses. In addition to large deletions, the small mutational events observed were predominantly single base pair deletions at the *gam* locus in both spontaneous mutants and mutants induced by asbestos (Table 1). There is evidence that deletions in the *gam* gene not only inactivate the *gam* gene but also interfere with the translation of the *redBA* gene, leading to functionally inactivate *gam* and *redBA* genes (Masumura et al. 2003). It should be noted that the maximum size of deletions detectable by the Spi^−^ assay is 9.6 kb. However, deletions extending into regions adjacent to the transgene concatemer are not detected, as two intact cos sites are required for the packaging of a single λ vector. Our present study indicated that the maximum deletion generated by chrysotiles in the *gpt* delta transgenic mutation system were kilobase-sized intrachromosomal deletions, which were much smaller than our previous reports on megabase–sized multilocus deletions generated by asbestos in the human–hamster cells (Hei et al. 1992)*,* largely because of the nature of the model system.

Various *in vitro* and *in vivo* studies have indicated that oxyradicals are one of the key determinants of asbestos-induced mutagenesis and carcinogenesis (Shukla et al. 2003). Among the most biologically active oxyradicals (e.g., superoxide anions (O_2_·), hydroxyl radical (·OH), singlet oxygen (^1^O_2_), and hydroperoxy radical (HO_2_·), H_2_O_2_ is relatively long-lived and directly crosses cell membranes by simple diffusion (Root et al. 1975). There is evidence that H_2_O_2_ not only induces damage to DNA, causing single- and double-strand breaks, base loss, base substitution, and cross-linking, but also causes chromosome and chromatid aberrations (Mondello et al. 2002). Recently, 8-hydroxydeoxyguanosine, an oxidative DNA damage marker, has been detected in Big Blue *lacI* transgenic rats treated with asbestos (Unfried et al. 2002). In an effort to understand the molecular mechanisms involved in the intra-chromosomal deletions induced by chrysotile in the present model, we compared mutation patterns between chrysotile asbestos and ROS. In the absence of serum, H_2_O_2_ produced predominantly ·OH radicals in human fibroblast culture (Weitzman and Graceffa 1984). Our results showed that for chrysotile-induced λ mutants the ratios of mutants with large deletions were similar to those induced by H_2_O_2_ at equitoxic doses. From a mechanistic point of view, these data suggest that similar mutagenic mechanisms are involved between asbestos fibers and chemically generated ROS. Consistent with this possibility, large mutational events mediated by oxyradicals have been observed in the human–hamster, AS52, and L5178 systems (Fach et al. 2003; Lipinski et al. 2000; Xu et al. 2002).

DSBs are usually regarded as the most deleterious type of DNA damage, induced either by environmental stress, such as irradiation or oxidative stress by the stalling of DNA replication forks (O’Driscoll and Jeggo 2006). Inefficient or inaccurate repair can elevate the frequencies of deletion, amplification, and chromosomal translocation, leading to chromosomal instability and neoplastic transformation. There is evidence that survival fraction and DSB-repair efficiency are dramatically decreased by chrysotile asbestos in the DNA DSB repair deficient cells as compared with wild-type cells (Okayasu et al. 1999). A very early step in the response of mammalian cells to DNA DSBs is the phosphorylation of histone H2AX at serine 139 at the sites of DNA damage (Lowndes and Toh 2005). Using γ-H2AX as a biomarker for DNA DSBs, our data showed that the accumulation of γ-H2AX was greatly increased by chrysotile treatment in MEF cells, which was inhibited by concurrent treatment with catalase. These findings provided strong corroborating evidence of the DNA damaging effects of chrysotiles through the oxyradical pathway.

Chromosomal rearrangements have been closely associated with the progression and maintenance of cancer (Radford 2004). One of the major difficulties in detecting *in vivo* somatic mutations in chromosomal DNA is the lack of systems capable of identifying and isolating mutated genes with high efficiency. Spi^−^ selection based on deletions extending into or through both the *redBA* and *gam* genes is an efficient mutation assay system for detecting small to kilobase-sized deletions in different cells, organs, and tissues (Nohmi and Masumura 2004). Although during packaging, the individual genes and vectors are segregated from each other and assayed for mutation independently, the target genes in the *gpt* delta system are present in multiple copies in tandem arrays and amount to a potential target of approximately 3.8 Mb. In reality megabase deletions cannot be distinguished from kilobase deletions because of the size limitation of lambda phage to be packaged. Thus, it is likely that the deletions that are induced by asbestos fibers in the present study may include intergenic deletions whose sizes are > 10 kb. As gene mutation, mitotic recombination, chromosome loss, and interstitial deletion largely contribute to the development of malignancy, the establishment of the *gpt* delta transgenic mouse mutation model may provide novel, mechanistic information on asbestos-induced genotoxicity in the future.

## Figures and Tables

**Table 1:** Type of λ-phage mutants at *redBA/gam* loci either of spontaneous origin or induced by chrysotile treatments (1 μg/cm^2^) determined by multiplex PCR analyses and DNA sequencing.

Groups	Total no. of mutants	No. of mutants with base substitution	No. of mutants with 1-bp deletion	No. of mutants with > 2-bp and < 1-kb deletions	No. of mutants with > 2-kb deletion
Control	93	7 (8%)	68 (73%)	8 (8%)	10 (11%)
Chrysotile	74	5 (7%)	41 (56%)	10 (14%)	17 (23%)

**Table 2:** Mutant fractions of deletions involving the *redBA/gam* region and other smaller deletions including single base changes in either nontreated control cells or cells treated with chrysotile fibers (1 μg/cm^2^ for 24 hr).

	Control	Asbestos
Total mutant fraction at *redBA/gam* loci	4.69 × 10^−6^	11.4 × 10^−6^
Large deletions (> 2 kb)		
Mutant fraction	0.5 × 10^−6^	2.6 × 10^−6^
Increase above the control	1.0	5.2
Small deletions plus single base changes		
Mutant fraction	4.2 × 10^−6^	8.8 × 10^−6^
Increase above the control	1.0	2.1
